# 初治多发性骨髓瘤患者早期死亡预测模型的建立

**DOI:** 10.3760/cma.j.issn.0253-2727.2021.08.009

**Published:** 2021-08

**Authors:** 梦茹 田, 珮钰 杨, 婷婷 岳, 梦瑶 李, 颖杰 张, 梦雪 张, 立茉 张, 玉蓉 闫, 中丽 胡, 雅哲 杜, 昱瑛 李, 凤艳 靳

**Affiliations:** 吉林大学第一医院血液科，长春 130012 Department of Hematology, the First Hospital of Jilin University, Changchun 130021, China

**Keywords:** 多发性骨髓瘤, 早期死亡, 预后因素, 预测模型, Multiple myeloma, Early mortality, Risk factors, Predictive model

## Abstract

**目的:**

分析影响初治多发性骨髓瘤（NDMM）患者早期死亡（EM）的因素，建立其预测模型，以期识别EM风险。

**方法:**

回顾性分析2009年5月至2017年1月吉林大学白求恩第一医院收治的275例NDMM患者，对6个月（EM6）、12个月（EM12）及24个月（EM24）内死亡患者初诊时基线特征进行单因素分析，并根据多因素分析结果建立EM的预测模型。

**结果:**

本研究中EM6、EM12及EM24的发生率分别是5.5%、12.7%和30.2%；最常见的死亡原因为疾病复发/进展，在EM6、EM12及EM24中分别占60.0%、77.1%及84.3%。影响EM6的因素包括经白蛋白校正的血清钙（校正钙）>2.75 mmol/L和PLT<100×10^9^/L；影响EM12的因素包括年龄>75岁、国际分期系统（ISS） Ⅲ期、修订版国际分期系统（R-ISS） Ⅲ期、校正钙>2.75 mmol/L、血清肌酐>177 µmol/L、PLT<100×10^9^/L及骨髓浆细胞比例≥60%；影响EM24的因素中，除上述影响EM12的因素外，还包括男性和染色体核型1q+。多因素分析尚未发现EM6和EM12的独立预后因素。在EM24的多因素分析中，年龄>75岁、PLT<100×10^9^/L和染色体核型1q+是EM24的独立预后因素。根据Logistic回归系数赋分：年龄>75岁：1分；PLT<100×10^9^/L：2分；染色体核型1q+：1分，建立EM24预测模型，ROC曲线下面积为0.709（95%*CI* 0.626~0.793）。积分≥3分的患者24个月内死亡风险是0~2分患者的26倍，积分0~4分的NDMM患者中位总生存期分别为59、41、22、17.5及16个月（*P*<0.001）。

**结论:**

年龄>75岁、PLT<100×10^9^/L和染色体核型1q+为EM24的独立预后因素，依据上述变量构建的EM24预测模型有助于识别EM风险和预测生存，具有较好标准度与区分度。

## Abstract (en)

**Objective:**

To investigate risk factors for early mortality (EM) in patients with newly diagnosed multiple myeloma (NDMM) and to build an EM-predictive model.

**Methods:**

In a cohort of 275 patients with NDMM, risk factors for EM at 6, 12, and 24 months after diagnosis (EM6, EM12, and EM24, respectively) were determined to establish a model to predict EM.

**Results:**

The rates of EM6, EM12, and EM24 were 5.5%, 12.7%, and 30.2%, respectively. The most common cause for EM was disease progression/relapse, accounting for 60.0%, 77.1%, and 84.3% of EM6, EM12, and EM24, respectively. EM6 was associated with corrected serum calcium >2.75 mmol/L and platelet count <100×10^9^/L, whereas risk factors for EM12 included age >75 years, ISS Ⅲ, R-ISS Ⅲ, corrected serum calcium >2.75 mmol/L, serum creatinine >177 µmol/L, platelet count <100×10^9^/L, and bone marrow plasma cell ratio ≥ 60%. In addition to the risk factors for EM12, EM24 was also associated with male sex and 1q21 gain. By multivariate analysis, age >75 years, platelet count <100×10^9^/L, and 1q21 gain were independent risk factors for EM24 but there were no independent risk factors significantly associated with EM6 and EM12. Using a scoring system including these three risk factors, a Cox model for EM24 was generated to distinguish patients with low (score<3) and high (score ≥ 3) risk. The sensitivity and specificity of the model were 20.7% and 99.2%, respectively. Further, an internal validation performed in a cohort of 183 patients with NDMM revealed that the probability of EM24 in high-risk patients was 26 times higher than that in low-risk patients. Moreover, this model was also able to predict overall survival. The median overall survival of patients with scores of 0, 1, 2, 3, 4, and 5 were 59, 41, 22, 17.5, and 16 months, respectively.

**Conclusion:**

In the study cohort, the EM6, EM12, and EM24 rates were 5.5%, 12.7%, and 30.2%, respectively, and disease progression or relapse were main causes of EM. An EM24-predictive model built on three independent risk factors for EM24 (age>75 years, platelet count<100×10^9^/L, and 1q21 gain) might predict EM risk and overall survival.

随着新药、新疗法的应用，多发性骨髓瘤（MM）患者的生存显著延长[1]，然而，早期死亡（EM）阻碍着MM患者整体生存的进一步延长。EM的时间界定尚未统一，多数研究将24个月内死亡纳入EM范畴[2]–[5]。但不同的研究对EM的阈值设定不尽相同，其中还包括EM2、EM6和EM12[6]–[12]。目前常用的包括国际分期系统（ISS）分期、修订版国际分期系统（R-ISS）分期、国际骨髓瘤工作组（IMWG）2014/2016年危险分层标准[13]–[14]、美国梅奥诊所mSMART[15]等危险分层/预后评估体系有助于预测MM的总体生存（OS），但并不能准确预测EM。目前所建立的EM预测模型主要是基于前瞻性临床试验，由于严格的入排标准（根据不同的研究目的排除了老年、并发症、体能状态差或不适合移植的人群等）并不能完全反映临床实践[2],[5],[10]；且由于不同研究定义的EM阈值不同、纳入研究的人群不同，影响EM的原因也有所不同[4]–[12],[16]。本研究我们以本中心275例NDMM患者为研究对象，平行分析有无发生EM6、EM12和EM24患者初诊时的临床特征和细胞遗传学改变，筛选不同EM阈值的危险因素，并构建EM预测模型。

## 病例与方法

1．病例：筛查了2009年5月至2017年1月吉林大学白求恩第一医院收治的510例NDMM患者，其中275例患者符合本研究的纳入标准：诊断符合IMWG 2014年多发性骨髓瘤诊断标准[17]及2013年原发性浆细胞白血病诊断标准[18]；具有基线水平血清学、骨髓细胞形态学、FISH检测结果[包括1q、del（17p）、del（13q）、del（1p23）、t（4;14）、t（11;14）、t（14;16）]；接受至少3个疗程以上的治疗；具有完整的随访资料，且随访时间≥2年或发生结局事件。本研究未纳入接受3个疗程以下的患者，基于下述原因：接受3个疗程以下的患者共88例，其中84例（95.5%）未接受治疗（56例，63.6%）或仅接受了≤ 1个疗程治疗（28例，31.8%），4例接受了2个疗程治疗（4.6%），所有这些患者由于就诊晚，尚未开始或刚开始治疗，即因疾病进展迅速而死亡，主要原因为疾病本身导致的并发症。

2．细胞遗传学危险分层及衰弱评分：根据2016年IMWG细胞遗传学危险分层标准[14]，参考国内外研究的FISH阈值[19]–[21]，对高危细胞遗传学异常（HRCA）定义如下：1q+（cut-off值6.0%）、del（17p）（cut-off值20.0%）、t（4;14）（cut-off值7.3%）和t（14;16）（cut-off值7.0%），其他非HRCA包括del（13p）/13q14和t（11;14）。由于我院FISH检测指标中不包括t（14;20），且文献报道t（14;20）检出率≤1%[14],[22]，对统计分析结果影响较小，故该HRCA未纳入研究。老年MM的衰弱评分依据2015年IMWG老年衰弱评分标准[23]。

3．早期死亡的定义：根据一项基于临床试验的EM研究[2]，并结合其他报道[3]–[5]，本研究将生存时间小于24个月定义为EM24，并同时与生存时间小于6个月（EM6）和12个月（EM12）进行比较分析。

4．治疗及疗效评价：275例NDMM患者中，以蛋白酶体抑制剂为基础的治疗占58.2%（160/275），以免疫调节剂为基础的治疗占34.5%（95/275），以单纯化疗为主占7.3%（20/275）。其中12.0%（33/275）的患者接受自体干细胞移植（ASCT）。疗效判定依据IMWG 2016年的MM疗效评价标准[24]和2013年浆细胞白血病的评价标准[18]，包括严格意义的完全缓解（sCR）、完全缓解（CR）、非常好的部分缓解（VGPR）、部分缓解（PR）、微小缓解（MR）、疾病稳定（SD）和疾病进展（PD）。

5．随访：采用电话或查阅门诊、住院病历的方式进行随访。随访截止时间为2020年1月6日，中位随访34（2~123）个月。无进展生存（PFS）时间定义为MM确诊之日至PD、复发或死亡日期。总生存（OS）时间定义为MM确诊之日至死亡或随访截止日期。

6．统计学处理：应用SPSS 22.0、Graphpad Prism 5.0和R 4.0.4软件进行统计学分析。分类变量比较采用*χ*^2^检验或Fisher精确概率法。生存分析采用Kaplan-Meier绘制生存曲线，显著性检验采用Log-rank法。多因素分析应用Logistic回归[纳入性别、年龄、疾病分期、CRAB症状（高钙血症、肾功能不全、贫血、溶骨性病变）、LDH、PLT、骨髓浆细胞等临床特征及细胞遗传学结果]，*P*<0.05为差异具有统计学意义。应用ROC曲线计算曲线下面积，定义临界点（cut-off）以区分高、低风险类别。

## 结果

1．临床特征：本研究共纳入275例NDMM患者，中位年龄59（31~87）岁；男150例（54.5%）、女125例（45.5%）；IgG型109例（39.7%）、IgA型68例（24.7%）、IgD型16例（5.8%）、轻链型74例（26.9%）及不（寡）分泌型8例（2.9%）；原发性浆细胞白血病（pPCL）5例（1.8%）；ISS分期中Ⅰ~Ⅱ期130例（47.8%），Ⅲ期142例（52.2%）；R-ISS分期中Ⅰ~Ⅱ期146例（66.7%），Ⅲ期73例（33.3%）；经白蛋白校正的血清钙（简称校正钙）>2.75 mmol/L 54例（19.9%）、血清肌酐>177 µmol/L 63例（22.9%）、血红蛋白<100 g/L 189例（68.7%）、溶骨性改变236例（90.1%）；体能状况评分（ECOG-PS）：<2分198例（72.0%），≥2分77例（28.0%）；FISH检测细胞遗传学异常（CA），其中1q+占49.2%、del（17p）占12.3%、del（13q）/13q14占50.8%、del（1p23）占12.4%；IgH重排中t（4;14）、t（11;14）、t（14;16）分别占23.1%、19.2%和6.6%。

2．EM6、EM12和EM24的死亡原因及治疗对EM的影响：275例NDMM患者中EM6、EM12和EM24的发生率分别为5.5%（15/275）、12.7%（35/275）和30.2%（83/275）。83例NDMM患者在24个月内死亡，其中3例患者因失访而不能明确死亡原因。EM6中死亡原因包括：疾病复发/进展、原发难治性MM、感染、心力衰竭（继发心肌淀粉样变）、黏连性肠梗阻/不明原因，分别占60.0%、6.7%、13.3%、6.7%及13.3%。在EM12的死亡原因中，疾病复发/进展占77.1%、原发难治性MM占2.9%、感染占8.6%、心力衰竭（继发心肌淀粉样变）占5.7%及黏连性肠梗阻/不明原因占5.7%。在EM24中，疾病复发/进展占84.3%、原发难治性MM占1.2%、感染占3.6%、心力衰竭（继发心肌淀粉样变）占6.0%及黏连性肠梗阻/不明原因占4.9%。由此可见，6、12、24个月内死亡患者中疾病复发/进展是主要死亡原因，并随着生存时间的延长所占比例逐渐增加。在年龄>75岁的MM患者中12例发生EM24，均为衰弱患者，其中16.4%死于并发症，而83.4%死于疾病进展，6例因为耐受性差中断治疗后出现疾病进展。根据2015年IMWG老年衰弱评分标准，其中7例（58.3%）衰弱患者仅因为年龄≥80岁（衰弱评分2分）；3例衰弱患者因年龄>75岁且工具性日常生活能力（IADL）5分（衰弱评分2分）；1例年龄>75岁合并脑血管疾病所致偏瘫（衰弱评分2分）；1例年龄>75岁、日常生活能力（ADL）3分和IADL 3分（衰弱评分3分）。伴1q+的患者中EM24的发生率为40%。其中，5.6%死于并发症，而94.4%死于疾病进展。

本研究中275例患者主要接受以蛋白酶体抑制剂（160例，占58.2%）和免疫调节剂（95例，占34.5%）为基础的治疗，故进一步分析了这两种不同治疗方案对EM的影响，结果显示：蛋白酶体抑制剂治疗组EM6、EM12、EM24发生率分别为4.4%、10.6%、25.0%，免疫调节剂治疗组EM6、EM12、EM24的发生率分别为5.3%、12.6%、35.8%，两组间差异均无统计学意义（*P*值均>0.05）。

3．EM6、EM12和EM24的单因素分析：见表1。影响EM6的因素包括校正钙>2.75 mmol/L（*OR*＝3.266，95%*CI* 1.083~9.850，*P*＝0.036）和PLT<100×10^9^/L（*OR*＝3.561，95%*CI* 1.203~10.544，*P*＝0.027），影响EM12因素包括年龄>75岁（*OR*＝3.613，95%*CI* 1.275~10.239，*P*＝0.022）、ISS Ⅲ期（*OR*＝3.580，95%*CI* 1.563~8.203，*P*＝0.002）、R-ISS Ⅲ期（*OR*＝3.612，95%*CI* 1.481~8.807，*P*＝0.003）、校正钙>2.75 mmol/L（*OR*＝4.008，95%*CI* 1.876~8.562，*P*<0.001）、血清肌酐>177 µmol/L（*OR*＝2.245，95%*CI* 1.057~4.768，*P*＝0.032）、PLT<100×10^9^/L（*OR*＝3.056，95%*CI* 1.394~6.697，*P*＝0.004）及骨髓浆细胞比例≥60%（*OR*＝2.750，95%*CI* 1.315~5.750，*P*＝0.006），影响EM24的因素中，除上述影响EM12的因素外，还包括男性（*OR*＝1.730，95%*CI* 1.019~2.939，*P*＝0.041）及染色体核型1q+（*OR*＝2.152，95%*CI* 1.137~4.071，*P*＝0.018）。

**表1:** 影响初治多发性骨髓瘤早期死亡的单因素分析

因素	EM6		EM12		EM24	
	*OR*（95%*CI*）	*P*值	*OR*（95%*CI*）	*P*值	*OR*（95%*CI*）	*P*值
年龄>75岁	2.190（0.459~10.547）	0.278	3.613（1.275~10.239）	0.022	4.467（1.691~11.801）	0.001
男性	0.950（0.335~2.696）	0.923	1.481（0.713~3.076）	0.290	1.730（1.019~2.939）	0.041
ISS Ⅲ期	2.645（0.821~8.524）	0.092	3.580（1.563~8.203）	0.002	2.303（1.345~3.942）	0.002
R-ISS Ⅲ期	2.610（0.679~10.031）	0.164	3.612（1.481~8.807）	0.003	2.485（1.352~4.571）	0.003
校正钙>2.75 mmol/L	3.266（1.083~9.850）	0.036	4.008（1.876~8.562）	<0.001	2.246（1.213~4.158）	0.010
血清肌酐>177 µmol/L	1.741（0.572~5.297）	0.346	2.245（1.057~4.768）	0.032	2.089（1.164~3.750）	0.013
血红蛋白<100 g/L	3.102（0.684~14.060）	0.158	1.625（0.706~3.741）	0.250	1.650（0.920~2.961）	0.091
溶骨性改变	1.457（0.183~11.614）	1.000	1.882（0.424~8.351）	0.547	1.192（0.480~2.960）	0.705
ECOG-PS≥2分	1.775（0.610~5.166）	0.374	1.873（0.898~3.906）	0.091	1.369（0.781~2.400）	0.271
LDH≥220 U/L	2.047（0.641~6.532）	0.314	1.785（0.776~4.107）	0.169	1.634（0.865~3.086）	0.128
PLT<100×10^9^/L	3.561（1.203~10.544）	0.027	3.056（1.394~6.697）	0.004	4.146（2.160~7.960）	<0.001
血清白蛋白<35 g/L	2.684（0.739~9.743）	0.120	1.465（0.686~3.129）	0.322	0.806（0.476~1.364）	0.421
骨髓浆细胞比例≥ 60%	1.150（0.353~3.739）	0.763	2.750（1.315~5.750）	0.006	2.221（1.248~3.953）	0.006
染色体核型1q+	2.116（0.378~11.851）	0.439	2.074（0.787~5.466）	0.134	2.152（1.137~4.071）	0.018

注：EM6：6个月内早期死亡；EM12：12个月内早期死亡；EM24：24个月内早期死亡；ISS：国际分期系统；R-ISS：修订版国际分期系统；校正钙：经白蛋白校正的血清钙；ECOG-PS：美国东部肿瘤协作组体能状况评分

4．EM6、EM12和EM24的多因素分析：尚未发现EM6和EM12的独立预后因素。EM24的多因素分析中发现3个独立预后因素：年龄>75岁（*OR*＝5.957，95%*CI* 0.897~39.560，*P*＝0.065），PLT<100×10^9^/L（*OR*＝12.172，95%*CI* 3.410~43.445，*P*<0.001），染色体核型1q+（*OR*＝3.730，95%*CI* 1.553~8.961，*P*＝0.003）（表2）。

**表2:** 影响初诊多发性骨髓瘤24个月内早期死亡的多因素分析

因素	*OR*（95%*CI*）	*P*值
年龄>75岁	5.957（0.897~39.560）	0.065
男性	1.125（0.493~2.565）	0.780
ISS Ⅲ期	0.947（0.335~2.672）	0.918
校正钙>2.75 mmol/L	1.218（0.420~3.534）	0.717
血清肌酐>177 µmol/L	1.529（0.481~4.866）	0.472
血红蛋白<100 g/L	1.414（0.500~3.997）	0.513
溶骨性改变	1.704（0.355~8.175）	0.505
ECOG-PS≥2分	0.793（0.289~2.181）	0.654
LDH≥220 U/L	1.329（0.510~3.464）	0.561
血清白蛋白<35 g/L	0.525（0.207~1.333）	0.175
PLT<100×10^9^/L	12.172（3.410~43.445）	<0.001
骨髓浆细胞比例≥ 60%	1.319（0.535~3.250）	0.547
染色体核型1q+	3.730（1.553~8.961）	0.003

注：ISS：国际分期系统；校正钙：经白蛋白校正的血清钙；ECOG-PS：美国东部肿瘤协作组体能状况评分

5．EM24预测模型的建立：年龄>75岁、PLT<100×10^9^/L及1q+回归系数分别为1.344、2.256、1.065，赋分如下：年龄>75岁：1分，PLT<100×10^9^/L：2分，染色体核型1q+：1分。根据NDMM患者积分建模，模型曲线下面积为0.709（95%*CI* 0.626~0.793），提示区分度良好；同时绘制该预测模型的校准曲线（图1），提示校准度良好。积分≥3分的患者24个月内死亡风险是0~2分的26倍，0~4分的NDMM患者中位OS期分别为59、41、22、17.5及16个月，差异有统计学意义（*P*<0.001）（图2）。

**图1:**
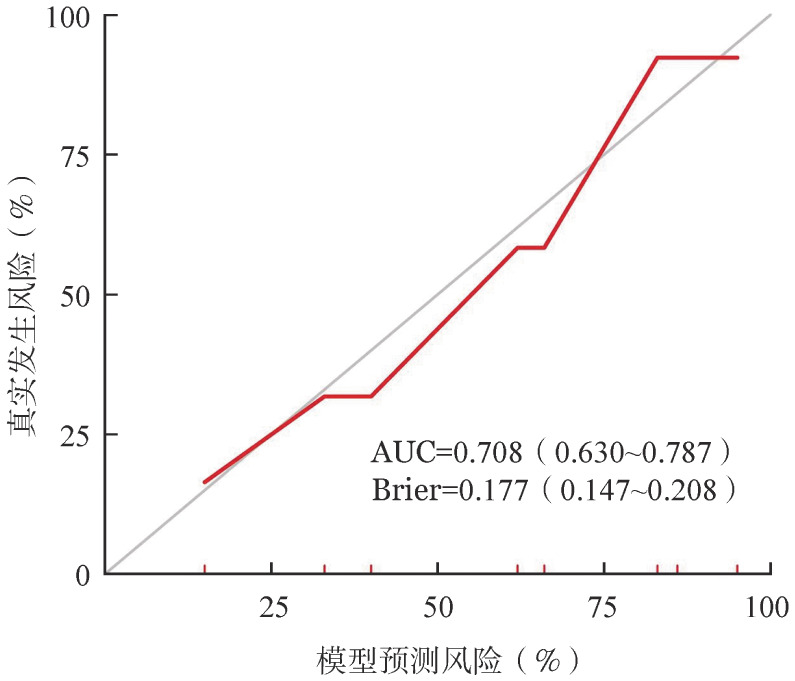
初诊多发性骨髓瘤24个月内早期死亡预测模型的校准曲线图

**图2:**
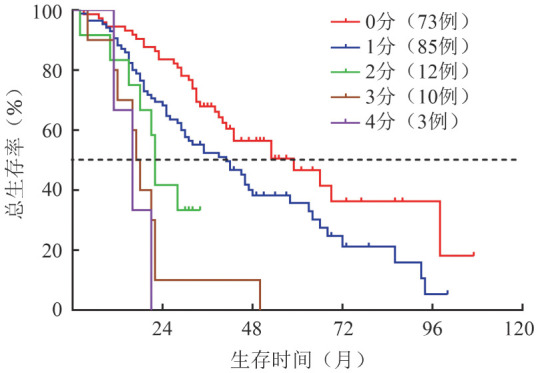
初诊多发性骨髓瘤24个月内早期死亡预测模型不同积分的生存曲线

## 讨论

本研究EM6、EM12及EM24的发生率分别是5.5%、12.7%和30.2%。最常见的死亡原因为疾病复发/进展，在EM6、EM12及EM24中分别占60.0%、77.1%及84.3%。单因素分析发现，高钙血症（校正钙>2.75 mmol/L）和PLT<100×10^9^/L是EM6的危险因素；而年龄>75岁、ISS Ⅲ期、R-ISS Ⅲ期、高钙血症、肾功能不全（血清肌酐>177 µmol/L）、PLT<100×10^9^/L及骨髓浆细胞比例≥60%是EM12的危险因素。除上述影响EM12的因素外，男性和染色体核型1q+是EM24的危险因素。本研究尚未发现影响EM6和EM12的独立危险因素，而年龄>75岁、PLT<100×10^9^/L和染色体核型1q+是EM24的独立危险因素。据此建立了预测EM24的预后模型，具有良好区分度与校准度。积分≥3分的患者24个月内死亡风险是0~2分的26倍，0~4分的NDMM患者中位OS期分别为59、41、22、17.5及16个月。

针对NDMM的前瞻性3期临床试验，由于严格的入排标准（根据不同的研究目的排除了老年、并发症、体能状态差或不适合移植的人群等），EM并不是常见的事件。纳入人群不同，研究方案不同，EM发生率差异性较大，例如IFM开展的比较硼替佐米联合地塞米松（VD）和减量的硼替佐米和沙利度胺联合地塞米松（VTD）方案治疗NDMM的研究中，EM发生率<5%，FIRST研究[25]中17% NDMM患者发生EM，而在MPT比较MP方案治疗老年NDMM的研究中，EM发生率高达25%。而观察性研究中，基于新药时代的美国Connect MM注册研究[10]中6.8% NDMM患者发生EM6，年龄>75岁，高ECOG和低EQ-5D评分是发生EM6的主要原因；而韩国一项多中心回顾性研究中，EM3、EM6、EM12发生率分别为3.1%、8.6%、13.8%，感染和合并症死亡是EM主要原因[26]，随着时间的延长，因疾病进展导致的EM增加。本研究中5.5%的NDMM患者发生EM6，低于上述两项研究EM6的发生率，可能由于本研究选择诱导治疗超过3个周期以上的NDMM病例，排除了诊断初期由于发生感染等并发症死亡的患者。而西班牙一项注册研究中所有NDMM的EM12的发生率高达31.9%。如果排除尚未治疗NDMM患者，EM12发生率为28.6%。其中，ISS分期、丙型肝炎病毒感染是EM12的独立危险因素[16]。本研究中EM12的发生率为12.7%，与韩国的研究结果相似，其中，年龄>75岁、ISS Ⅲ期、R-ISS Ⅲ期、高钙血症、肾功能不全、PLT<100×10^9^/L及骨髓浆细胞比例≥60%是EM12的高危因素。

目前仅有几项针对EM24的研究，由于纳入人群不同，其发生率和预后模型相差甚远。如IFM2005-01研究中EM24发生率为9.1%，LDH、ISS Ⅲ期和不良细胞遗传学异常[t（4;14）和/或17p-]是EM24独立危险因素[5]。该预测模型的建立仅纳入适合移植的NDMM患者。而西班牙一项基于GEM2005和GEM2010两项临床试验主要是研究年龄≥65岁老年NDMM因疾病进展发生EM24的危险因素，13.8%的老年NDMM因疾病进展发生EM24[2]，因此该预测模型仅适合预测老年NDMM因疾病进展发生EM24的风险。丹麦一项基于大剂量化疗联合ASCT治疗NDMM的登记注册研究中，EM24发生率仅9.6%，提示包括ASCT的系统治疗明显降低EM风险，特别是因为疾病进展发生EM的风险[4]。北京协和医院针对生存期短于24个月的NDMM的回顾性研究中41.2%的NDMM患者发生EM24[3]，主要死亡原因为疾病进展（67.12%）。与之相似，本研究中EM24发生率30.2%。其中，84.3%是由于疾病复发/进展。除上述影响EM12的高危因素外，男性和携带染色体核型1q+的NDMM患者EM24发生风险增高。而年龄>75岁、PLT<100×10^9^/L和染色体核型1q+是EM24的独立危险因素。

MM是一种老年性疾病，根据美国国家癌症研究所的SEER数据库统计显示MM患者中位发病年龄为69岁，其中年龄>65岁的占63.5%[27]。老年MM，尤其是衰弱老年MM预后不良，主要由于治疗耐受性差，治疗相关毒性事件及停药发生率明显增加[23],[28]。多项研究显示>75岁是EM的高危因素[2],[10],[12]。本研究中年龄>75岁不仅是EM12的高危因素，也是EM24独立预后因素。染色体核型1q+是MM最常见的细胞遗传学异常改变：国外报道30%~40%的NDMM携带1q+[13],[29]–[30]，国内报道接近50%的NDMM伴1q+[31]，明显高于西方人群。多项研究证实携带1q+的NDMM患者PFS和OS期明显缩短[31]–[33]。本研究首次报道染色体核型1q+是EM24独立危险因素。携带1q+的NDMM患者常具有疾病晚期、高肿瘤负荷、易伴其他高危细胞遗传学改变的特点[31],[33]–[34]。进一步分析显示：伴1q+的患者中EM24发生率为40.0%。其中，5.6%死于并发症，而94.4%死于疾病进展。提示对于伴1q+的患者应密切关注疗效，采取个体化治疗取得较深缓解，延缓疾病进展发生。越来越多的研究发现，伴血小板减少的NDMM患者OS期明显缩短[35]–[36]，易发生EM[8],[10]–[11],[26]。但不同的研究中设定的血小板阈值不同。本研究显示PLT<100×10^9^/L不仅是EM12的危险因素，也是EM24的独立危险因素。对于血小板数量在EM的预后价值及其阈值还需进一步研究确定。

综上，本研究平行分析了EM6、EM12和EM24的发生率及高危因素，据此建立了预测EM24的预后模型。通过建立EM预测模型有助于对NDMM患者客观、有效地进行EM风险评估，进而针对高风险患者制定个体化治疗方案降低EM发生，延长总生存时间。值得注意的是，尽管多数研究将24个月内死亡纳入EM范畴[2]–[5]，但目前对EM的界定尚未统一，本研究比较分析了EM6、EM12和EM24相关的危险因素，发现其不尽相同，提示明确和统一EM定义在这一研究领域中的重要性。本预后模型的建立基于单中心回顾性研究，故有待多中心研究及前瞻性登记注册临床研究进一步验证。
